# Effects of the COVID-19 pandemic on diet and physical activity and the possible influence factors among Saudi in Riyadh

**DOI:** 10.3389/fnut.2022.1029744

**Published:** 2022-10-20

**Authors:** Lujain Abdulaziz Almousa, Reham Ibrahim Alagal

**Affiliations:** Department of Health Sciences, College of Health and Rehabilitation Sciences, Princess Nourah Bint Abdulrahman University, Riyadh, Saudi Arabia

**Keywords:** COVID-19, physical activity, diet, pandemic, nutrition

## Abstract

**Background/Aim:**

The COVID-19 pandemic has been spreading throughout the world, having a significant impact on people’s lifestyles and health through social isolation and home confinement. The purpose of this study is to look into the impact of COVID-19 on diet and physical activity, as well as the possible influence factors, among ≥ 13-year-olds in Riyadh, Saudi Arabia.

**Materials and methods:**

In the present study data were collected from 2,649 participants *via* an online survey. The Google online questionnaire was available from April 23 to May 6, 2020. During the COVID-19 lockdown, the survey asked respondents about their demographic characteristics (gender, age, education, economic income, and occupation), anthropometric data, physical activity, and diet habits.

**Results:**

The study included 2,649 respondents, with 23.3% being male and 76.7% female. The majority of them were in good health and ranged in age from 21 to 29 years. 31% of those polled were overweight, and 14.3% were obese. The majority of respondents have a bachelor’s degree, diploma, or the equivalent, and a monthly family income of ≤ 25.000 SR. Those who were following a healthy diet (32.3%) were unable to maintain it during confinement, with males being affected more than females (42.7%, 29.3%, respectively, *P* = 0.004), and those most impacted were aged 21–29 years (38.0%, *P* = 0.046). Furthermore, 59.5% of males significantly failed to continue exercising during confinement compared to females who exercised consistently (*P* = 0.01). In terms of age, females aged less than 40 increased their exercise rate by about 23.4%, while males aged 40 and up decreased their exercise rate by 25.7% (*P* = 0.000). Moreover, 40.5% of the subjects’ weight increased, according to the findings. However, there was no significant effect on body mass index, despite the fact that 51% of participants were overweight or obese.

**Conclusion:**

The data showed that the COVID-19 lockdown had a negative impact on maintaining a healthy diet (*p* = 0.023*) and physical activity (*p* = 0.000^**^).

## Introduction

Coronavirus disease-19 (COVID-19) is a public health crisis that originated in Wuhan, China (1). The Coronavirus disease 2019 (COVID-19) pandemic has the potential to cause considerable morbidity and mortality, affecting the people and health care systems globally (2). COVID-19 has been classified as a pandemic, and health organizations have advised people throughout the world to stay inside and limit their contact with the outside world (3). In Kingdom of Saudi Arabia the total number of confirmed cases with COVID-19 rose from one on 2 March 2020 to 101,914 on 7 June, representing an average of 1,039 new cases per day. Despite the increase in the number of newly confirmed daily cases of COVID-19, the number of reported daily active cases started to stabilize after 2 months from the start of the pandemic in the country, and the overall recovery rate was 71.4% and the mortality rate decreased by 6.4% (COVID-19 Dashboard: Saudi Arabia). COVID-19 was more common among adults and males compared to other demographic groups (4). Therefore, the Kingdom of Saudi Arabia (K.S.A.) announced a series of restrictions due to the increasing number of COVID-19 cases with little mortality in the capital. These constraints have resulted in a significant shift in people’s daily lives, and most organizations have requested that their employees work from home using virtual meeting rooms. Restaurants, shops, and malls were closed beginning March 23rd. Schools and universities were closed, and direct instruction was replaced with virtual education; flights, intercity travel, and public gatherings were also prohibited. Supermarkets and bakeries remained open, providing residents with essential supplies (5).

Lifestyle factors are habitual and routine behaviors that can have an impact on physical and mental health. Dietary habits, physical activity, sleeping patterns, and smoking are all important factors in maintaining a healthy body (6). A local study carried out by Al-Musharaf et al. (7) looked at how quarantine affected Saudi women’s lifestyle, weight, and sleeping habits. They concluded that there was a significant impact on the lifestyle behaviors of young Saudi women, particularly weight change. Individuals who are food insecure are also known to consume a lot of carbohydrates and fats while eating very few fruits and vegetables (8). Such changes in eating habits caused by boredom, anxiety, stress, depression, or food insecurity may increase the risk of developing obesity and comorbidities like cardiovascular disease and type 2 diabetes, which may be more harmful than COVID-19 (9). During the COVID-19 pandemic, increased macronutrient intake may result in the replacement of important micronutrients required for good health (10). A healthy diet also has a positive effect on the immune system, as it helps to activate immune cells. Nutrition deficiencies, such as a lack of vitamin A, B6, B12, E, iron, zinc, and protein, weaken immune functions, making people more susceptible to infections (11). In addition, unhealthy foods that are rich in fats and sugars are harmful to the body and lower immunity (12). Many restaurants and coffee shops have closed since the lockdown began, making it more difficult to go out to eat with friends (13, 14). Many people find it difficult to control themselves when passing a fast-food restaurant, eating in a school cafeteria, or even purchasing food at work (15). Hence, quarantine provides an excellent opportunity for them to change their unhealthy eating habits and begin leading healthy lifestyles by meeting daily nutrition requirements (11, 16).

Physical activity can help people’s immune systems by relieving stress, including the stress caused by COVID-19 outbreak concerns. Guidelines recommend that people engage in 150–300 min of physical activity per week, and they can get some of this exercise by doing housework. Setting weekly goals for physical activity at home will also aid in the reduction of feelings of isolation (17). Inactivity affects up to 31% of 15-year-olds, and unhealthy lifestyles kill 3.2 million people yearly (18). Despite this, physical inactivity is now the fourth leading cause of death worldwide, with a higher percentage than obesity (19). Fitness centers have been closed during this pandemic (17), and people are advised not to engage in outdoor activities such as walking, jogging, and exercising in public parks and gymnasiums. Their restrictions further altered their daily routines (3). Staying at home, where there is less movement and more time spent sitting in front of screens, can lead to other health problems for people who engage in regular physical activity (20). While attempting to avoid a coronavirus encounter, a variety of home-based exercises can be performed. People can get exercise by doing things like walking around the house or taking the stairs up and down the building’s floors (21). Just because people’s movements are restricted does not mean they should limit their physical activities or abandon all forms of exercise. Everyone should set goals for themselves to be physically active for a certain amount of time each day (3). Given the importance of diet and physical activity in the prevention and treatment of many chronic diseases, assessing the effects of the Corona pandemic on physical activity and diet is critical. Taking into account the presumed benefits and drawbacks of a lockdown due to the COVID19 outbreak, the current study was designed to investigate the impact of COVID-19 on diet and physical activity in the Saudi population in Riyadh.

## Materials and methods

### Subjects

The Riyadh city was chosen to conduct the study because it is the most populous city in the Kingdom of Saudi Arabia, with a population that is a mix of all Saudi cities. Therefore, random sample collection leads to differences in cultural, social, and economic factors. Furthermore, Riyadh was under full lockdown at the time, while the majority of the cities were under partial lockdown. Riyadh has a population of 7,231,447 people, 72% of whom are over the age of 14 (22). As a result, a sample size of at least 2,400 people was estimated to evaluate the chosen variables, assuming a 5% margin of error and a response proportion of 50% with a 95% confidence level. This survey was open to all people aged 13 and up who lived in Riyadh, Kingdom of Saudi Arabia, and were invited to voluntarily participate in the survey by responding to an online questionnaire. People from other cities (a total of 629 participants) were excluded. The results of the first 2,649 responses to an online survey on lifestyle behaviors during home confinement due to the COVID-19 pandemic were reported. Because of the need to reduce contact between people due to home confinement due to the COVID-19 pandemic, an online questionnaire was chosen.

### Study questionnaire

Ten nutrition experts reviewed and validated the initial questionnaire and were given a week to provide feedback. Specific modifications and amendments were made and amended based on their feedback, such as correcting linguistic errors, rewording some questions, and adding questions to describe changes in the participants’ nutritional intake. The validated online survey was launched on 14 April 2020, tested for 1 week, and then made available online on 23 April 2020. The Google online questionnaire was available from April 23 to May 6, 2020. During the COVID-19 lockdown, it was administered in Arabic and included 45 questions about the respondents’ socio-demographic characteristics (gender, age, education, economic income, occupation), health, mental wellbeing, mood, life satisfaction, and lifestyle behaviors (physical activity, diet, sleep), as well as anthropometric data. Questions about “before” and “during” confinement periods were presented in sequence to be answered. Given the many questions, the current paper focuses on diet and exercise before and during confinement. Participants were also asked to self-report their height and weight values so that their body mass index (B.M.I.) and weight status (underweight/normal/weight/overweight/obese) could be calculated (23).

### The ethical statement, data privacy, and consent of participation

The study was conducted according to the Declaration of Helsinki statement on research using human participation. The protocol and the consent form were approved (IRB log number : 20-0250) by the Institutional Review Board at Princess Nourah bint Abdulrahman University. The informed agreement was taken online from each participant prior to starting the survey and after having been informed about the aim of the study. During the informed consent process, survey participants were assured that all data would be used only for research purposes. Participants’ answers are anonymous and confidential according to Google’s privacy policy.^1^ Participants were not permitted to provide their names or contact information. Additionally, participants were able to withdraw and leave the questionnaire at any stage without explanation before the submission process; if doing so, their responses would not be saved. Answers were saved only by clicking on the provided “submit” button. By completing the survey, participants acknowledged their voluntary consent to participate in this study.

### Survey promotion and data collection

The survey was uploaded and shared on the Google online survey platform. A link to the electronic survey was distributed *via* a range of methods: invitation *via* e-mail, Facebook™, WhatsApp™ and Twitter™, Instagram™, and Snapchat™. The survey included an introductory page describing the background and the aims of the survey and ethical information for participants. By clicking the survey link, participants were automatically directed to the data sharing and privacy policy to be accepted before starting. The present study focuses on the first two thousand responses (i.e., 2,649 participants), which were reached on 6 May 2020, approximately 1 week after survey dissemination.

### Data analysis

For statistical analysis, the statistical package for social sciences (SPSS Inc., Chicago, IL, U.S.A.) was used (Statistical Package for Social Sciences software, version 22). Categorical variables were presented using frequencies and percentages. Continuous variables with an approximate normal distribution are represented by means and standard deviations, while continuous variables with a skewed distribution are represented by medians and their interquartile ranges. The chi-square test for categorical variables and one-way analysis of variance for continuous variables were used to examine group differences. To compare the means of two groups, the Student’s *t*-test was used.

## Results

### Demographic characteristics of the participants

According to Table 1, there were 2,649 respondents in this study, with 23.3% being male and 76.7% being female. The majority of them were found to be in good health, ranging in age from 21 to 29 years. 31% were obese, and 14.3% were overweight. The majority of those polled had a bachelor’s degree, diploma, or the equivalent, and a monthly family income of 25.000 SR.

**TABLE 1:** Demographic characteristics of the participants.

Variables		Frequency	%
Gender	Male	618	76.7%
	Female	2,031	23.3%
Age (years)	13–20	627	23.7%
	21–29	769	29.0%
	30–39	494	18.6%
	40–49	402	15.2%
	50–59	260	9.8%
	≥ 60	97	3.7%
B.M.I.	Underweight	197	7.4%
	Normal	1,102	41.6%
	Overweight	821	31.0%
	Obese	378	14.3%
	Severely obese	106	4.0%
	Morbid obesity	45	1.7%
Health state	Healthy	2,143	80.9%
	With cancer	12	0.5%
	With heart disease	18	0.7%
	With diabetes	107	4.0%
	With blood pressure	109	4.1%
	With kidney disease	9	0.3%
	With respiratory diseases	98	3.7%
	With immunodeficiency diseases	19	0.7%
	Other diseases	134	5.0%
Level of education	Postgraduate	251	9.5%
	Bachelor’s degree, diploma, or the equivalent	1,720	64.9%
	High school graduate or lower	678	25.6%
Employment status	Employed for wages (Government)	662	25.0%
	Employed for wages (Private sector)	393	14.8%
	Self-employed	98	3.7%
	Out of work/Unemployed/Retired	533	20.1%
	A student	907	34.2%
	Other	56	2.1%
Family monthly income	≥ 10.000 SR	593	22.4%
	From 10.000 to 14.999 SR	504	19.0%
	From 15.000 to 19.999 SR	485	18.3%
	From 20.000 to 24.999 SR	333	12.6%
	≤ 25.000 SR	734	27.7%

### The impact of COVID-19 lockdown on diet and physical activity among participants

**FIGURE 1:**
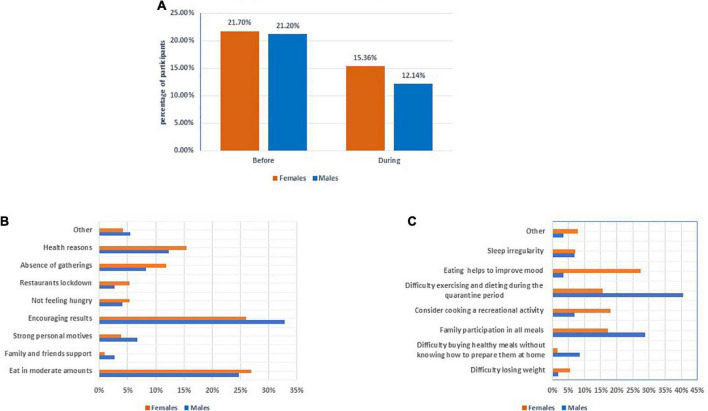
Gender differences in dieting during the confinement period. **(A)** Follow the diet according to gender before and during the confinement period. **(B)** Causes to continuing following healthy diet during the confinement period. **(C)** Causes to stopping following healthy diet during the confinement period.

Before the confinement period, 21.6% of the participants committed to a healthy diet for a period ranging from a month or less (40.0%) to a year or more (21.2%), with no gender differences (Table 2). The primary reasons for following a healthy diet were encouraging results (27.1%), the ability to eat rationally (26.4%), maintaining health (14.7%), and the absence of social gatherings (11.1%). During the confinement period, one-third (32.3%) of people who were following a healthy diet were unable to maintain it, with males being affected more than females (42.7%, 29.3%, respectively) (*P* = 0.004) (Figure 1), and those most impacted were aged 21–29 years (38.0%) (*P* = 0.046) (Supplementary Table 1). Males’ inability to stick to a healthy diet was attributed to difficulties exercising and maintaining a diet during the lockdown period (40.7%), family participation in all meals (28.8%), difficulty purchasing healthy meals without knowing how to prepare them at home (8.5%), and sleep irregularity (6.8%). However, these reasons differ significantly among women who were unable to adhere to a healthy diet (*P* = 0.000), with the most common reasons being eating helps to improve mood (27.3%) and considering cooking a recreational activity during the quarantine period (18.0%) (Figure 1).

**TABLE 2:** Dieting before and during the confinement period.

		Dieting before the confinement period	Dieting during the confinement period
No (78.4%)		2,077 (78.4%)	2,262 (85.39%)
Yes (21.6%)	> 1 month	229 (40.0%)	387 (14.61%)
	1 month to > 2 months	108 (18.9%)	
	2 months to > 3 months	50 (8.6%)	
	3 months to > 5 months	23 (4.0%)	
	5 months to > 12 months	41 (7.2%)	
	≤ 12 months	121 (21.2%)	

**FIGURE 2:**
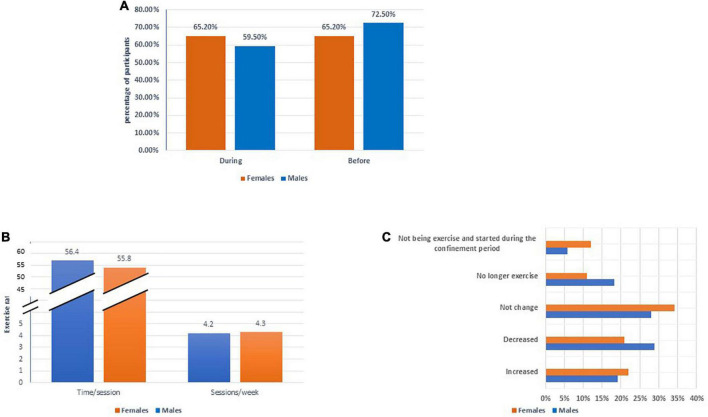
Gender differences in exercising during the confinement period. **(A)** Exercising according to gender before and during the confinement period. **(B)** Cause to stopping following healthy diet during the confinement period. **(C)** Changes in exercise rate during the confinement period.

Overall, the majority of participants exercised before and during confinement (66.9%, 63.9%, respectively) for an average of 4 times per week and 90 min per session (before confinement), with males (72.5%) exercising more than females (65.2%) (*P* = 0.001) (Table 3 and Supplementary Table 2). During the confinement period, 32.8% of participants did not change their exercise rate, compared to 22.7% who decreased it and 21.3% who increased it. However, 59.5% of males significantly failed to continue exercising during confinement compared to females who exercised consistently (*P* = 0.01) (Figure 2). In terms of age, females aged less than 40 years increased their exercise rate by about 23.4%, while males aged 40 and up decreased their exercise rate by 25.7% (*P* = 0.000) (Supplementary Table 2).

**TABLE 3:** Exercising before and during the confinement period.

		Exercising before the confinement period	Exercising during the confinement period
No		887 (33.1%)	956 (36.1%)
Yes	Once/week	1,772 (66.9%)	197 (10.5%)
	3 times/week		386 (20.7%)
	4 times/week		231 (12.4%)
	5 times/week		551 (29.5%)
	6 times/week		196 (10.5%)
	7 times/week or more		132 (7.1%)

### Effect of Education and income on a diet and exercising before and during the confinement period

In general, the education level of the participants did not affect their diet or exercise habits (Table 4). However, the reasons for success differ according to educational level among those who were able to adhere to a healthy diet, with the ability to eat in moderate quantities being the most important factor among high school graduates or lower and bachelor’s degree, diploma, or the equivalent holders (34.9, 26.2%, respectively) while postgraduates (34.0%) were motivated by the encouraging results (*P* = 0.017). Furthermore, the rate of exercising during the confinement period was significantly affected by educational level (*P* = 0.000), with an increase of 23.0% among those who received a bachelor’s degree, diploma, or the equivalent and 14.3% among those who received a high school diploma or lower starting to exercise, with no significant difference between a set of education and either the mean of the number of exercise sessions per week or the time of a session. There was no significant relationship between monthly family income and eating a healthy diet and exercising before or during confinement. However, there was a positive relationship between the rate and average time of exercise, as well as the income level, during the confinement period compared to the previous period (*P* = 0.003; 0.018, respectively) (Table 5).

**TABLE 4:** Education, diet, and exercise before and during the confinement period.

Variable		Category	High school or lower	Bachelor’s/diploma	Postgraduate	Chi-square	*P*
Diet	Before the confinement period	No	79.8%	78.5%	73.7%	4.07	0.131
		Yes	20.2%	21.5%	26.3%		
	Period of diet	> 1 month	41.6%	39.6%	39.4%	6.18	0.800
		1 month to > 2 months	17.5%	18.7%	22.7%		
		2 months to > 3 months	5.8%	9.2%	12.1%		
		3 months to > 5 months	3.6%	4.3%	3.0%		
		5 months to > 12 months	8.0%	6.5%	9.1%		
		≤ 12 months	23.4%	21.7%	13.6%		
	During the confinement period	No	36.5%	31.2%	30.3%	1.44	0.487
		Yes	63.5%	68.8%	69.7%		
	Causes to continuing following healthy diet during the confinement period	Eat in moderate amounts	34.9%	26.2%	12.8%	30.2	0.017**
		Family and friends support	–	2.0%	–		
		Strong personal motives	7.0%	3.2%	6.4%		
		Encouraging results	18.6%	29.0%	34.0%		
		Not feeling hungry	11.6%	3.2%	4.3%		
		Restaurants lockdown	4.7%	5.2%	4.3%		
		Absence of gatherings	4.7%	11.9%	19.1%		
		Health reasons	14.0%	14.7%	17.0%		
		Other	4.7%	4.8%	2.1%		
	Causes to stopping following healthy diet during the confinement period	Difficulty losing weight	5.9%	3.4%	5.3%	5.4	0.980
		Difficulty buying healthy meals without knowing how to prepare them at home	5.9%	2.6%	5.3%		
		Family participation in all meals	11.8%	23.9%	26.3%		
		Consider cooking a recreational activity	15.7%	14.5%	10.5%		
		Difficulty exercising during the quarantine period	23.5%	23.9%	21.1%		
		Eating helps to improve mood	21.6%	18.8%	21.1%		
		Sleep irregularity	7.8%	6.8%	5.3%		
		Other	7.8%	6.0%	5.3%		
Exercise	Before the confinement period	No	36.1%	32.4%	29.9%	4.39	0.111
		Yes	63.9%	67.6%	70.1%		
	During the confinement period	No	36.4%	36.3%	33.9%	0.60	0.74
		Yes	63.6%	63.7%	66.1%		
	Rate during the confinement period	Increased	18.4%	23.0%	17.9%	34.0	0.000**
		Decreased	19.9%	23.7%	23.1%		
		Not change	36.6%	30.5%	38.2%		
		No longer exercise	10.8%	13.1%	14.3%		
		Not being exercise and started during the confinement period	14.3%	9.8%	6.4%		
	Number of sessions per week (mean ± SD)*	Session/week	4.18 ± 1.80	4.22 ± 1.60	4.27 ± 1.58		0.831
	Period of sessions (mean ± SD)*	Minutes/session	0.90 ± 0.67	0.95 ± 0.66	0.93 ± 0.66		0.447

*Values are means ± S.D. of three samples.

**Mean values significantly different at level *p* ≤ 0.05.

**TABLE 5:** Income, diet, and exercising before and during the confinement period.

Variable		Category	≥10000 SR	From 10,000 to 14,999 SR	From 15,000 to 19,999 SR	From 20,000 to 24,999 SR	≤25,000 SR	Chi-square	*P*
Diet	Before the confinement period	No	80.8%	77.4%	79.2%	77.2%	77.2%	3.33	0.505
		Yes	19.2%	22.6%	20.8%	22.8%	22.8%		
	Period of diet	> 1 month	47.4%	36.0%	41.6%	44.7%	34.7%	16.1	0.710
		1 month to > 2 months	16.7%	15.8%	23.8%	14.5%	21.6%		
		2 months to > 3 months	7.9%	10.5%	9.9%	5.3%	9.0%		
		3 months to > 5 months	4.4%	6.1%	3.0%	2.6%	3.6%		
		5 months to > 12 months	6.1%	9.6%	5.0%	9.2%	6.6%		
		≤ 12 months	17.5%	21.9%	16.8%	23.7%	24.6%		
	During the confinement period	No	36.8%	35.1%	33.7%	30.3%	27.5%	3.43	0.488
		Yes	63.2%	64.9%	66.3%	69.7%	72.5%		
	Causes to continuing following healthy diet during the confinement period	Eat in moderate amounts	31.4%	18.7%	33.8%	28.3%	23.5%	43.75	0.081
		Family and friends support	2.9%	1.3%	1.5%	–	0.8%		
		Strong personal motives	2.9%	6.7%	7.4%	5.7%	1.7%		
		Encouraging results	18.6%	30.7%	29.4%	24.5%	30.3%		
		Not feeling hungry	12.9%	2.7%	4.4%	3.8%	3.4%		
		Restaurants lockdown	7.1%	9.3%	–	3.8%	4.2%		
		Absence of gatherings	8.6%	9.3%	7.4%	9.4%	16.8%		
		Health reasons	11.4%	18.7%	8.8%	17.0%	16.8%		
		Other	4.3%	2.7%	7.4%	7.5%	2.5%		
	Causes to stopping following healthy diet during the confinement period	Difficulty losing weight	4.7%	7.7%		13.0%		26.76	0.351
		Difficulty buying healthy meals without knowing how to prepare them at home	2.3%	2.6%	3.0%	13.0%	2.0%		
		Family participation in all meals	18.6%	25.6%	15.2%	17.4%	24.5%		
		Consider cooking a recreational activity	16.3%	10.3%	24.2%	8.7%	12.2%		
		Difficulty exercising during the quarantine period	25.6%	12.8%	30.3%	21.7%	26.5%		
		Eating helps to improve mood	18.6%	23.1%	18.2%	13.0%	22.4%		
		Sleep irregularity	4.7%	10.3%	6.1%	4.3%	8.2%		
		Other	9.3%	7.7%	3.0%	8.7%	4.1%		
Exercise	Before the confinement period	No	34.4%	33.5%	33.8%	30.3%	32.6%	1.86	0.762
		Yes	65.6%	66.5%	66.2%	69.7%	67.4%		
	During the confinement period	No	39.3%	37.3%	34.4%	31.2%	36.0%	6.95	0.139
		Yes	60.7%	62.7%	65.6%	68.8%	64.0%		
	Rate during the confinement period	Increased	16.2%	19.4%	24.3%	23.7%	23.7%	34.49	0.005**
		Decreased	22.9%	23.8%	24.7%	21.9%	20.6%		
		Not change	32.9%	31.2%	29.7%	36.0%	34.3%		
		No longer exercise	15.0%	13.5%	10.3%	10.2%	12.7%		
		Not being exercise and started during the confinement period	13.0%	12.1%	10.9%	8.1%	8.7%		
	Number of sessions per week (mean ± SD)	Session/week	3.98 ± 1.69	4.19 ± 1.63	4.19 ± 1.63	4.17 ± 1.72	4.44 ± 1.58		0.003**
	Period of sessions (mean ± *SD*)	Minutes/session	0.96 ± 0.70	0.88 ± 0.61	0.85 ± 0.64	0.98 ± 0.70	0.99 ± 0.65		0.018*

*p < 0.05, **p < 0.01.

### Effect of gender and age on body mass index during the confinement period

**FIGURE 3:**
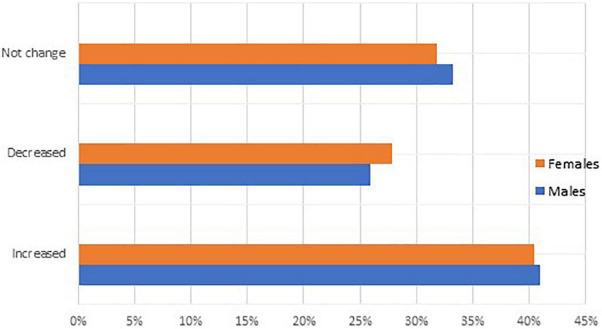
BMI during the confinement period.

The confinement period had a general effect on all participants’ B.M.I., with 40.5% experiencing an increase and 26.3% experiencing a decrease. However, with respect to gender the results showed no significant differences between males and females (Figure 3). COVID-19 has a significant effect on diet and exercise, as both have decreased their use (*p* = 0.000^**^) (*p* = 0.023*, respectively) (Supplementary Table 3). The participants aged 30–39 years had the highest rates of weight maintenance (34.4%), while the elderly aged 60 and over had the highest rates of weight gain (53.6%), and the participants aged 21–29 years had the highest rate of weight loss (28.6%) (Table 6). Overall, 41.6% of participants had a normal body mass index (B.M.I.), 31.0% were overweight, and 14.3% were obese. There were no discernible gender differences (Table 1).

**TABLE 6:** B.M.I. for age groups before and during the Confinement Period.

Variable	Category	Age (years)							
		13–20	21–29	30–39	40–49	50–59	≥ 60 years	Chi-square	*P*
Change in weight	Not change	31.4%	33.7%	34.4%	28.6%	31.9%	27.8%	16.12	0.096
	Increased	42.4%	37.7%	38.1%	41.3%	43.1%	53.6%		
	Decreased	26.2%	28.6%	27.5%	30.1%	25.0%	18.6%		

## Discussion

The findings of this study are to show and understand how the pandemic and the enforced lockdown had an effect on the dietary health and physical activity of the Saudi in Riyadh. Riyadh was chosen as the study’s location because it is the most populous city in the Kingdom of Saudi Arabia, with a population drawn from all Saudi cities. As a result, cultural, social, and economic differences result from random sampling. The lockdown had a negative impact on participants who claimed to have followed a healthy diet prior to the COVID-19 lockdown. Moreover, males were affected more than females, and those most affected were aged 21–29. This could be due to high levels of anxiety during quarantine as a result of COVID-19 news, as well as working from home, which resulted in different stress-related eating habits (24) and eating out of boredom (25). Furthermore, spending a lot of time in front of the television can increase the number of meals and the amount of food consumed (26). Di Renzo et al. (24) discovered that 37% of the Italian population studied changed their eating habits during the peak of COVID-19, which can weaken the immune system. Consumption of foods high in saturated fatty acids reduces the adaptive immune system (27), whereas eating healthy foods improves the immune system (28). However, some studies contend that obesity and an unhealthy diet are linked to low education and economic levels, as these people may have little nutrition education and may be unable to afford healthy food (29, 30). On the contrary, the current study found no significant effect of education level or family income on practicing and maintaining a healthy diet during the COVID-19 lockdown. Most likely, because the majority of the participants in this study were educated and may have knowledge of nutritional facts, their monthly family income remained unchanged from before the lockdown.

The results of this survey revealed that the main motivators for eating a healthy diet were to promote better health (19.2%) and lose weight (17.7%), which is consistent with the findings of a study that found that health motives have a significant impact on healthy dietary patterns and weight control (31). In terms of health promotion, encouraging a wide variety of healthy foods, particularly fruits and vegetables, can help one avoid health problems (32, 33). Furthermore, several studies have revealed that those who are concerned about their appearance or health are more likely to exercise restraint when eating (34, 35). In this regard, the primary goal of dieting is weight loss (29). However, in the current study, we discovered that weight loss as a result of dieting encouraged 27.1% of the participants to stick to their diet, whereas eating rationally inspired 26.4% of them to continue dieting. It appears that when weight loss is observed, it motivates people on diets to continue and limit calorie intake (36), and losing a significant amount of weight helped to improve self-esteem (37), which led to diet success and achievement of the desired weight level (38). Eating in moderation, that is, considering the balance and variety of foods consumed (39, 40), is important for more than just avoiding weight gain. It is a good eating strategy to avoid overeating (41) and balance eating for health and pleasure (42).

The main reasons for diet failure were also considered in this study: 23.5% of those who did not continue their diet found it difficult to follow a healthy diet and exercise during COVID-19 quarantine. Most of these were male, and 20.9% of participants who failed their diet because they shared all main meals with their family were males, and 19.8% of those who broke their diet discovered that eating helps improve their mood during curfews, which affect women more. Lockdowns were imposed in a number of countries to limit the spread of COVID-19, which included closing gyms and open places where people go for activities and exercise, limiting physical activity to what could be done indoors (25). Furthermore, grocery shopping constraints hampered food storage and influenced daily activities and eating attitudes (24). One change in eating habits that occurred during quarantine concerns eating the main meal as a family. According to research, sharing meals with the family has been linked to a healthy diet. However, if the family meal is not nutritious, it may result in weight gain (43). Living with a family that consumes high-calorie foods is frequently linked to diet failure (29). Thus, to promote healthy diets and avoid unhealthy ones, it is necessary to educate families about nutrition (44).

Another source of concern revealed by the current study’s findings is that COVID-19 curfews have a significant effect on exercise. Physical inactivity is a global cause of death (19). Physical activity boosts the immune system and aids in the inhibition of proinflammatory cytokines (45, 46). Furthermore, exercise aids in the prevention of respiratory illnesses and influenza (17). During the lockdown, however, the number of males aged 40 and up who exercised decreased, while females continued to exercise. When compared to the time before COVID-19, 22.7% of those who continued to exercise decreased their exercise rate, while 21.3% increased their exercise rate. Furthermore, during the COVID-19 quarantine, physical activity was reduced, and sedentary behavior increased in more than 40% of the Italian university population (47). These findings are similar to those of another Chinese study, which discovered that the pandemic had a negative impact on Chinese adults’ physical activity levels, with 64% of participants being physically inactive during the COVID-19 pandemic (48). In a Dutch study (49), low physical activity was observed in approximately half of the sample. Furthermore, physical activity decreased for 43.0% of Greek participants (50). Physical activity may have been reduced during COVID-19 curfews because more people stayed at home, increasing the amount of time spent sitting and doing various screen activities (3). Indeed, additional findings have revealed that the COVID-19 quarantine had a negative impact on all types of physical activity, with time spent inactive increasing by 28% (25). Curfews, on the other hand, do not have to mean limiting physical activity because there are numerous ways to be active indoors (51). According to one study, many athletes increased their training frequency (24).

The current study discovered that education level and family income did not affect exercise habits. Many studies, on the other hand, suggest that high school and college students are irregularly active (52, 53). A study conducted on adults aged 30–70 in Saudi Arabia discovered that education level and income are positively associated with physical activity (54). Anxiety and boredom during the COVID-19 quarantine have led to increased eating and decreased activity, which has led to weight gain for many people (55). Previous research found that people were well-informed about the pandemic. Physical activity (P.A.) decreased significantly during the lockdown, particularly among those who were already inactive (56, 57). According to Di Renzo et al. (24), 8.3% of the study’s participants gained weight during the lockdown, 40% gained minor weight, and 37.3% remained stable. According to data from a cross-sectional study in Spain, 52.7% of subjects aged 16 and up gained weight during confinement (58). In a Poland study, 29.9% of those polled reported gaining weight during quarantine (59). In a cross-sectional, online Ibero-American survey of adult men and women aged 18 years from four Latin American countries (Argentina, Brazil, Mexico, and Peru), it was estimated that half of the participants gained weight during the COVID-19 lockdown (60). Gallè et al. (61) investigated the effects of the COVID-19 pandemic in people over the age of 65 in southern Italy and reported that the weight of 66.3% of participants with males recorded higher percentage than females. According to this study, during the COVID-19 quarantine, 32.1% of the participants’ weight did not change, 40.5% gained weight, and 27.3% lost weight. It was discovered that the weight of participants aged 60 years in Riyadh increased by 53.6%, decreased by 27.8%, and remained unchanged by 18.6%.

In addition, a previous study found that 0.4% of people over the age of 65 in southern Italy were underweight, 23.6% were normal weight, 49.7% were overweight, and 26.3% were obese. Body Mass Index participants who reported being overweight were mostly females with a high level of education who lived in a community setting (62). While the results of this study show that the quarantine had no significant effect on the participants’ B.M.I. 41.6% had a normal B.M.I., 31% were overweight, 14.3% were obese, 4.0% were severely obese, and 1.7% was morbidly obese. In this regard, a study on Saudi adults found that 40.9% had a normal B.M.I., 25% were overweight, and 17% were obese (63). The current study shows an increase in overweight, which can be attributed to high diet consumption and lower physical activity due to COVID-19 isolation. Another study in Hail of 5,000 Saudi adults found that 37% were overweight and 15% were obese (64). Obesity is associated with a lack of physical activity and eating high-calorie foods in Saudi Arabia (62). Physical inactivity is linked to weight gain and exercise helps regulate body weight (7). Physical activity was increased by 3.1% of participants, 23 (5.3%) male and 9 (1.5%), decreased by 58.2%, 185 (42.6%) male, and 421 (69.4%) female, and remained unchanged in 38.7%, 226 (52.1%) male and 177 (29.2%) female (61).

Overall, the current study’s data showed that the COVID-19 lockdown had a negative effect on physical activity (*p* = 0.023*) and, more significantly, on maintaining a healthy diet (*p* = 0.000^**^). Males’ inability to stick to a healthy diet was most affected by difficulty exercising and maintaining a diet during the lockdown period (40.7%) and family participation in all meals (28.8%). According to the findings, the main motivators for eating a healthy diet were to promote better health (19.2%) and to lose weight. Approximately 32.3% of people who were following a healthy diet were unable to keep it up during the quarantine period, males (42.7%) were more affected than females (29.3%) (*P* = 0.004), and people aged 21–29 years were the most affected (38.0%) (*P* = 0.046). Furthermore, 59.5% of males were significantly less likely to continue exercising than females (*P* = 0.01), with females aged 40 years increasing their exercise rate by 23.4% while males decreased their exercise rate by 25.7% (*P* = 0.000). Furthermore, 40.5% of the subjects’ weight increased, according to the findings. However, there was no significant impact on B.M.I., with 51% of participants being overweight or obese.

The authors are well aware of some limitations in this study, as this survey was conducted *via* the internet. The most significant limitations were that it did not include the most important population group, children and infants aged ≤ 13 years, a low rate of participation of older adults aged 50 years, and participants’ caloric intake was not measured. Furthermore, the anthropometric measurements were self-reported and could be measured inaccurately. Furthermore, there is a higher proportion of women than men. Finally, due to the COVID-19 restriction, conducting person-to-person questionnaires or interviews was difficult. However, in addition to limiting contact with people during the pandemic, this method has a number of advantages, including high-accuracy questionnaire preparation, ease of distribution to the targeted on a large scale, access to and collection of data and results in record time, and at the lowest cost, ease of processing and analyzing data quickly and with high accuracy, avoiding the embarrassment that researchers may face during personal interviews, and avoiding the embarrassment that researchers may face during personal interviews, obtaining more reliable results due to the participants’ freedom in their responses, free of any influence on their opinions.

## Conclusion

The results of this survey clarify that the participants were varied with respect to age and socioeconomic characteristics. COVID-19 pandemic has affected both the diet and physical activity of the study population and was found to be declining as the COVID-19 pandemic is ongoing. Males’ inability to stick to a healthy diet was most affected by difficulty exercising and maintaining a diet during the lockdown period, as well as family participation in all meals. Generally, females continued to follow a healthy diet and exercise during the lockdown, while males did not. Weight gain was observed in 40.5% of the population. Surprisingly, more than half of the participants were overweight or obese, and education level and income were not associated with the ability to maintain a healthy diet and stay active. Educational programs should be implemented in the community to improve diet and activity routines as preventive measures against the effects of COVID-19 and contribute to public health improvement and protection from health risks.

## Data availability statement

The original contributions presented in this study are included in the article/Supplementary material, further inquiries can be directed to the corresponding author/s.

## Ethics statement

The studies involving human participants were reviewed and approved by (IRB log number: 20-0250) by the Institutional Review Board at Princess Nourah Bint Abdulrahman University. The patients/participants provided their written informed consent to participate in this study.

## Author contributions

LA and RA: conceptualization, methodology, writing—original draft preparation and review, and editing. LA: software, visualization, formal analysis, resources, and funding acquisition. RA: validation, investigation, data curation, supervision, and project administration. Both authors have read and agreed to the published version of the manuscript.

## Footnotes

1
https://policies.google.com/privacy?hl=en
