# Supplementary material for: Effects of the COVID-19 pandemic on diet and physical activity and the possible influence factors among Saudi in Riyadh
Source: Front Nutr. 2022 Oct 20;9:1029744. doi: 10.3389/fnut.2022.1029744 (PMC9630832; doi:10.3389/fnut.2022.1029744)
Supplement: Supplementary file 1 [file Table_1.docx]

**Supplementary Table 1. The effect of age on diet**

| Variable | category | 13 - 20 years | 21 - 29 years | 30 - 39 years | 40 - 49 years | 50 - 59 years | 60 years and over | Chi-square | P |
| --- | --- | --- | --- | --- | --- | --- | --- | --- | --- |
| following healthy diet before the confinement period | No | 84.4% | 78.4% | 77.1% | 72.9% | 74.2% | 80.4% | 23.8 | 0.000** |
|  | Yes | 15.6% | 21.6% | 22.9% | 27.1% | 25.8% | 19.6% |  |  |
| Period of being on diet | less than month | 46.9% | 40.4% | 34.5% | 41.3% | 38.8% | 31.6% | 38.6 | 0.04* |
|  | From month to less than 2 month | 19.4% | 22.9% | 23.9% | 11.9% | 14.9% | 5.3% |  |  |
|  | From 2 month to 3 month | 4.1% | 7.2% | 8.8% | 13.8% | 10.4% | 10.5% |  |  |
|  | From 3 month to less than 5 month | 2.0% | 4.2% | 3.5% | 5.5% | 6.0% | - |  |  |
|  | From 5 month to less than 1 year | 7.1% | 3.6% | 8.0% | 11.9% | 9.0% | - |  |  |
|  | from year or more | 20.4% | 21.7% | 21.2% | 15.6% | 20.9% | 52.6% |  |  |
| following healthy diet during the confinement period | No | 33.7% | 38.0% | 34.5% | 25.7% | 31.3% | 5.3% | 11.31 | 0.046* |
|  | Yes | 66.3% | 62.0% | 65.5% | 74.3% | 68.7% | 94.7% |  |  |
| main reason of diet commitment | I eat rationally | 21.5% | 26.7% | 27.4% | 19.5% | 39.1% | 38.9% | 87.51 | 0.000** |
|  | I have a lot of support from family / friends | 3.1% | 1.0% | 2.7% | - | - | - |  |  |
|  | I have a strong cause for weight loss (for example: an impor | 4.6% | 5.0% | 6.8% | 3.7% | 2.2% | - |  |  |
|  | The results of the diet were clear and encouraging | 26.2% | 29.7% | 27.4% | 36.6% | 13.0% | 11.1% |  |  |
|  | I did not feel hungry | 10.8% | 5.9% | 4.1% | 3.7% | 2.2% | - |  |  |
|  | Closing restaurants helped me stick to the diet | 10.8% | 6.9% | 4.1% | 2.4% | - | - |  |  |
|  | The absence of family meetings or with friends, which were d | 7.7% | 14.9% | 12.3% | 12.2% | 8.7% | - |  |  |
|  | Health reasons | 4.6% | 5.0% | 12.3% | 19.5% | 32.6% | 50.0% |  |  |
|  | Other | 10.8% | 5.0% | 2.7% | 2.4% | 2.2% | - |  |  |
| main reason of failure to commitment the diet | Difficulty losing weight | - | 4.5% | 5.1% | 3.7% | 9.5% | - | 37.9 | 0.34 |
|  | Difficulty obtaining diet foods without knowing how to prepa | 9.1% | 3.0% | 2.6% |  | 4.8% | - |  |  |
|  | Share all main meals with the family | 3.0% | 16.7% | 33.3% | 25.9% | 28.6% | 100.0% |  |  |
|  | Cooking is an entertaining activity during the social spacin | 27.3% | 12.1% | 12.8% | 7.4% | 14.3% | - |  |  |
|  | Difficulty exercising during a social spacing due to COVID-1 | 15.2% | 31.8% | 15.4% | 29.6% | 19.0% | - |  |  |
|  | Eating helps me improve my mood | 27.3% | 22.7% | 12.8% | 18.5% | 14.3% | - |  |  |
|  | Irregular sleep | 9.1% | 7.6% | 7.7% | 7.4% |  | - |  |  |
|  | Other | 9.1% | 1.5% | 10.3% | 7.4% | 9.5% | - |  |  |
