# Supplementary material for: Effects of the COVID-19 pandemic on diet and physical activity and the possible influence factors among Saudi in Riyadh
Source: Front Nutr. 2022 Oct 20;9:1029744. doi: 10.3389/fnut.2022.1029744 (PMC9630832; doi:10.3389/fnut.2022.1029744)
Supplement: Supplementary file 2 [file Table_2.docx]

**Supplementary Table 2. The effect of age on exercise**

| Variable | category | 13 - 20 years | 21 - 29 years | 30 - 39 years | 40 - 49 years | 50 - 59 years | 60 years and over | Chi-square | P |
| --- | --- | --- | --- | --- | --- | --- | --- | --- | --- |
| Exercising before the confinement period | No | 36.7% | 33.0% | 34.2% | 29.6% | 30.8% | 25.8% | 9.12 | 0.104 |
|  | Yes | 63.3% | 67.0% | 65.8% | 70.4% | 69.2% | 74.2% |  |  |
| Exercising during the confinement period | No | 33.8% | 36.2% | 36.6% | 38.8% | 36.5% | 35.1% | 2.8 | 0.73 |
|  | Yes | 66.2% | 63.8% | 63.4% | 61.2% | 63.5% | 64.9% |  |  |
| The rate of exercise during the confinement period | Increased | 23.3% | 24.8% | 22.1% | 15.2% | 16.5% | 15.5% | 89.02 | 0.000** |
|  | Decreased | 17.5% | 22.5% | 24.7% | 25.9% | 25.4% | 25.8% |  |  |
|  | Has not changed | 34.6% | 29.4% | 30.4% | 35.6% | 35.4% | 41.2% |  |  |
|  | I no longer exercise | 8.1% | 12.2% | 13.8% | 16.9% | 15.0% | 14.4% |  |  |
|  | I didn't do sports and now I started | 16.4% | 11.1% | 9.1% | 6.5% | 7.7% | 3.1% |  |  |
|  | | | | | | | | | |
|  | age | 13 - 20 years | 21 - 29 years | 30 - 39 years | 40 - 49 years | 50 - 59 years | 60 years and over | f | P |
| Exercise sessions/week | Mean | 4.17 | 4.32 | 4.17 | 4.16 | 4.08 | 4.51 | 1.18 | 0.316 |
|  | Std. Deviation | 1.83 | 1.48 | 1.61 | 1.66 | 1.70 | 1.69 |  |  |
| Exercise time/session | Mean | 0.98 | 1.03 | 0.91 | 0.80 | 0.82 | 0.87 | 5.87 | 0.000** |
|  | Std. Deviation | 0.67 | 0.73 | 0.59 | 0.59 | 0.56 | 0.72 |  |  |
