# Supplementary material for: Effects of the COVID-19 pandemic on diet and physical activity and the possible influence factors among Saudi in Riyadh
Source: Front Nutr. 2022 Oct 20;9:1029744. doi: 10.3389/fnut.2022.1029744 (PMC9630832; doi:10.3389/fnut.2022.1029744)
Supplement: Supplementary file 3 [file Table_3.docx]

Supplementary 3. exercising , diet and BMI Before and During COVID-19

| Variable | category | Before COVID-19 | During COVID-19 | Chi-square | p |
| --- | --- | --- | --- | --- | --- |
| exercising | No | 33.1% | 36.1% | 5.21 | 0.023* |
|  | Yes | 66.9% | 63.9% |  |  |
| diet | No | 78.4% | 85.4% | 43.58 | 0.000** |
|  | Yes | 21.6% | 14.6% |  |  |
| BMI | Underweight | 7.4% | 6.3% | 3.36 | 0.645 |
|  | Normal | 41.6% | 41.9% |  |  |
|  | Overweight | 31.0% | 31.3% |  |  |
|  | Obese | 14.3% | 14.3% |  |  |
|  | Severely Obese | 4.0% | 4.5% |  |  |
|  | Morbid obesity | 1.7% | 1.6% |  |  |
